# Editorial: Adhesive hydrogels: design, fabrication, and bio-applications

**DOI:** 10.3389/fbioe.2023.1290228

**Published:** 2023-09-21

**Authors:** Qihui Zhou, Yong Liu, Mohamed Sayed Hasanin, Tengbo Yu

**Affiliations:** ^1^ School of Rehabilitation Sciences and Engineering, University of Health and Rehabilitation Sciences, Qingdao, China; ^2^ Wenzhou Institute, University of Chinese Academy of Sciences, Wenzhou, China; ^3^ Cellulose and Paper Department, National Research Centre, Cairo, Egypt; ^4^ Qingdao Hospital, Qingdao Municipal Hospital, University of Health and Rehabilitation Sciences, Qingdao, China

**Keywords:** adhesive hydrogels, biomaterials, biomedical applications, disease treatment, tissue repair and regeneration

Adhesive hydrogels have been appealing as emerging biomedical materials for high demand for fundamental research and practical biomedical applications owing to inherent self-adhesiveness, flexible structure, dynamic mechanical properties, and near-physiological environment (Hao et al., 2022; Li et al., 2022; Mei et al., 2022). The integrated adhesion between the hydrogel interface and surrounding tissues is one of the most critical interactions that offer overall robustness and reliability during specific environment (Duan et al., 2023b; Han et al., 2023; Zeng et al., 2023). Based on their unique (bio) physicochemical features above, adhesive hydrogels have been applied in numerous indications, including implant scaffolds in tissue engineering, hemostasis, mucosal adhesives to extend the administration site time, and bio-adhesives in place of wounds to reduce infection (Hao et al., 2022; Ko and Liao, 2023; Zhao et al., 2023). In addition, therapeutic cells and drug molecules can be transported by adhesive hydrogels and released at the injury site to enhance efficacy (Duan et al., 2023a; Zhang et al., 2023). Adhesive hydrogels perform suitably in clinical investigations for soft tissue regeneration, anti-exudation, medicine distribution, and other applications. This Research Topic focuses on the recent advances regarding adhesive hydrogels and their biomedical applications, aiming to provide a reference for researchers in related fields. We have collected nine original research articles.

These groundbreaking materials, the result of meticulous design considerations and inventive processes for fabrication, have the potential to redefine bio-applications and change medical technology. This takes us on an expedition into the fascinating world of adhesive hydrogels, exploring their sophisticated production processes, varied design, and an incredible range of revolutionary bio-application they provide. In original research articles on this Research Topic about the regulation of biomaterial base Adhesive hydrogel for tissue repair and regeneration, Zhu et al. developed an adhesive hydrogel for loading of conditioned medium (CM) based on Gel-MA (CM/Gel-MA), which was greatly adhesive and favors the stable retention of CM. CM/Gel-MA boosted the activity of endometrial stromal cells (ESCs), promoting cell proliferation and reducing α-SMA, collagen I, CTGF, E-cadherin, and IL-6 expression to reduce the inflammatory response and inhibit fibrosis. CM/Gel-MA hydrogel prevented uterine adhesion (IUA) through physical barriers from adhesive hydrogel and functional promotion from CM. Qu et al. prepared a hyaluronic acid (HA) and *Bletilla striata* polysaccharide (BSP) based dissolving microneedle patches (MNs) to mediate mesoporous polydopamine nanoparticles (MPDA) loaded with Triamcinolone acetonide (TA) as the transmucosal delivery system to treat oral mucositis (OM). A@MPDA-HA/BSP MNs possessed well-arranged microneedles, good penetration efficiency, quick dissolution in 3 min, excellent biocompatibility, and anti-inflammatory effects. MNs decreased oral ulcer area and inflammatory factor levels such as TNF-α and CD31. Yang et al. introduced an innovative method using oxidized regenerated cellulose and fibrin glue to prevent air leakage following lung segmentectomy. Their approach revealed efficacy in an *ex vivo* porcine lung model, suggesting a potential alternative to existing methods. Li et al. proposed a novel hydrogel-based method for enumerating fetomaternal hemorrhage, a critical factor in preventing newborn hemolytic diseases. Their approaches offer a clinically applicable prenatal diagnosis technique, addressing the limitations of traditional methods.

In contrast, biomaterials have been commonly used in antimicrobial/cancer applications. For antimicrobial research, Wei et al. addressed the challenges of cytotoxicity in silver nanoparticles by developing a coating technique using polydopamine. This approach enhanced antibacterial properties and biocompatibility, presenting a promising route for diverse medical applications. In addition, Hajizadeh et al. presented a solution for capturing haemin using albumin-based cryogels, showcasing higher binding capacity and reaction rates compared to conventional approaches. Their work has potential implications for preventing alloimmunization. Wang et al. explored shape-memory fibers for tissue regeneration, particularly in the osteogenic differentiation of stem cells. They demonstrate enhanced shape recovery and mechanical properties by manipulating fiber structure and programming parameters, offering new avenues for biomaterial applications.

In conclusion, the original research papers in this Research Topic indicate adhesive hydrogels broadly impact tissue healing, regeneration, drug delivery, and infection control. Researchers from Sweden and China cooperate to usher in a new biomedical era, stressing the significance of the accomplishment globally. These groundbreaking findings demonstrate the dynamic potential of adhesive hydrogels, revolutionizing healthcare and allowing improved cures and treatments.
